# Synthesizing genetic sequential logic circuit with clock pulse generator

**DOI:** 10.1186/1752-0509-8-63

**Published:** 2014-05-28

**Authors:** Chia-Hua Chuang, Chun-Liang Lin

**Affiliations:** 1 Department of Electrical Engineering, National Chung Hsing University, Taichung 402, Taiwan, ROC

**Keywords:** Genetic oscillator, Clock signal, Logic circuit, Optimization

## Abstract

**Background:**

Rhythmic clock widely occurs in biological systems which controls several aspects of cell physiology. For the different cell types, it is supplied with various rhythmic frequencies. How to synthesize a specific clock signal is a preliminary but a necessary step to further development of a biological computer in the future.

**Results:**

This paper presents a genetic sequential logic circuit with a clock pulse generator based on a synthesized genetic oscillator, which generates a consecutive clock signal whose frequency is an inverse integer multiple to that of the genetic oscillator. An analogous electronic waveform-shaping circuit is constructed by a series of genetic buffers to shape logic high/low levels of an oscillation input in a basic sinusoidal cycle and generate a pulse-width-modulated (PWM) output with various duty cycles. By controlling the threshold level of the genetic buffer, a genetic clock pulse signal with its frequency consistent to the genetic oscillator is synthesized. A synchronous genetic counter circuit based on the topology of the digital sequential logic circuit is triggered by the clock pulse to synthesize the clock signal with an inverse multiple frequency to the genetic oscillator. The function acts like a frequency divider in electronic circuits which plays a key role in the sequential logic circuit with specific operational frequency.

**Conclusions:**

A cascaded genetic logic circuit generating clock pulse signals is proposed. Based on analogous implement of digital sequential logic circuits, genetic sequential logic circuits can be constructed by the proposed approach to generate various clock signals from an oscillation signal.

## Background

Synthetic biology is an emerging interdisciplinary research field, which concentrates on understanding the behaviors of biological system from system-level as well as creating an artificial genetic circuit based on the principles of systems biology, mathematics and engineering [1-4]. Analogous to an electronic circuit, the synthetic genetic circuit also includes some standard biological components to assemble the biochemical process of living organisms and achieve specific functionality. Based on a bottom-up approach, more complicated bio-computing modules can be expected to perform more complex functions via integrating a variety of biological devices, like very-large-scale integration circuits in electronics. By using mathematical models to capture the quantitative and qualitative characteristics of biological systems, the customized genetic circuits with specific functions can be designed from the system perspective [5-8]. For drug development and disease treatment, synthetic biology brings a useful and rapid direction through inserting the designed genetic circuits into the host cells to improve or modify the disease state of organisms. In addition, there are still potential applications in biofuels, biotechnology, bioremediation, and bioenergy remained to be developed.

Inspired by electronic circuits, several synthetic genetic circuits have recently been created, such as toggle switch, genetic oscillator, pulse generator, genetic counter, logic evaluator, sensor, filter, and cell-cell communicator. The former twos are based on protein-protein interaction without any external input to control their behaviors. Toggle switch applies two repressor genes repressing each other to cause bi-stable phenomenon, like as a memory device [9]. By cascading odd number of repressor genes in the cycle chain, a genetic oscillator can be synthesized to generate a stable oscillation signal in the protein response and applied in the control of dosage of drugs, or regarded as a synchronous mechanism for cell-cell communication [10-13]. A pulse generator generates an instantaneous stimulating signal and then resets to the original state by using time difference between the input and the corresponding delayed signals [14,15]. If the input signal is a periodic clock signal, then a clock pulse signal can be synthesized. Biosensor and filter are designed to detect the concentrations of specific molecular signal and range [8].

Boolean logic gate is an essential unit of a computer in digital logic circuits. To bring the insight of digital logic circuit design in electronic systems into biological systems, the more complicated bio-computing processes can be easily constructed by combining a variety of genetic logic gates. The genetic logic gates constructed are based on different genetic transcriptional reactions to express various logic behaviors [16-22]. To use genetic components such as promoter, ribosomal binding site (RBS), repressor/activator genes and reporter gene, genetic logic gates with different logical operations have been assembled, such as NOT, Buffer, AND, OR, XOR. Through synchronous cascades of these genetic logic gates based on the topology of digital logic circuits, more complicated genetic logic circuits can be synthesized, such as multiplexer, half adder, combinational logic circuits, memory, and sequential logic circuits [23-30]. A genetic sequential logic circuit works with a counters, which is composed of some basic devices such as SR latch and flip-flop, has been developed in [25,29].

In biological systems, there are different rhythm frequencies depending on cell types. A 12-hour rhythm has been recently found in the mouse liver. For this reason, there are many engineered approaches proposed to synthesize the specific oscillation signals. In [31], the frequency-doubling oscillation can be constructed by using Fourier theory. A genetic circuit with multiple functions is designed to synthesize the oscillation signal with half original frequency [32]. Another aspect is to use regulated protein to control the transcription and degradation rates of target gene in an existing network structure [33-35]. A robust synthetic genetic circuit is designed based on *H*_∞_ control theory by regulating degradation rates of mRNAs and proteins in stochastic perturbational environments [33,34]. For cell-cell communication, synchronized genetic circuit designs are proposed to synchronize a population of oscillation signals [36,37]. To construct a promoter-RBS library from microarray data and find suitable promoter-RBS components, a robust genetic circuit has been theoretically realized in the genetic systems by a systematic approach [38,39].

This paper proposes an artificial genetic sequential logic circuit with a function of frequency divider based on the periodic oscillation signal from a repressilator and analogous topology of the digital logic circuits in electronics. The proposed genetic sequential logic circuit is triggered by a clock pulse signal to generate a clock signal whose frequency is an inverse integer multiple to the genetic oscillator. Similar to an electronic waveform-shaping circuit, a genetic waveform-shaping circuit constructed by several genetic Buffers in series is designed, which regulates time duration of logic high/low levels of an oscillation signal in the basic sinusoidal cycle and reshapes the oscillation signal into a pulse-width-modulated (PWM) signal with different duty cycles by regulating the different threshold levels of the Buffer. The PWM signal can be regarded as a pulse signal with the frequency is coherent to that of the genetic oscillator. The clock pulse signal is served as the rising or falling triggered edges of a clock signal with base frequency. In the digital logic theory, Karnaugh map is applied to determine the input signals of the rising or falling edge-triggered genetic JK flip-flops in each level [40]. A synchronous genetic counter circuit is triggered by the clock pulse signal to realize the genetic clock with its frequency is an inverse integer multiple to the genetic oscillator.

For our proposed genetic pulse generator design, the periodic property of genetic oscillator is considered and the clock pulse signal is generated by utilizing the existing synthetic genetic oscillator constructed by three repressor genes which repress each other in the closed loop. Different from the genetic counter circuit design [25,29], we introduce a generalized form based on the topology of digital logic circuits for synthesizing a clock signal with an inverse multiple of clock frequency to the genetic oscillator. The major advantage of the proposed approach is that it is easy to construct complex genetic sequential logic circuits via bottom-up approach with less computational time. Simulation results *in silico* show performance of the synthesizing clock pulse signal, and the clock signal with double, triple, quadruple basal periods while operating at the same genetic oscillator.

## Methods

### Dynamic model of synthetic genetic logic circuits

By applying mathematical models to describe the biochemical reactions of genetic systems, a synthetic genetic circuit with a specific function can be synthesized from the system's perspective.

Consider the dynamic model of the synthetic genetic logic circuit with *L* genes described by a class of nonlinear Hill differential equation [7]

(1) $$\begin{array}{l} {\dot{m}}_{i}=\alpha_{i}f_{i}\left(u\right)-\lambda_{i}m_{i}+\alpha_{i,0},\\ {\dot{p}}_{i}=\beta_{i}m_{i}-\gamma_{i}p_{i},i=1,\ldots ,L \end{array}$$

where *m*_*i*_ and *p*_*i*_ denote, respectively, concentrations of mRNA and protein for the gene *i*, *λ*_*i*_ and *γ*_*i*_ are, respectively, the degradation rates of mRNA and protein, *α*_*i*_ is the transcription rate of mRNA, *β*_*i*_ is the synthesis rate of protein, *α*_*i*,0_ is the basal production rate, *f*_*i*_(⋅) is the promoter activity function which describes the nonlinear transcriptional behavior and reflects the strength of the interaction between regulated protein and RNA polymerase (RNAp), and *u* is the concentration of transcription factor (TF) which is produced from other gene(s) or inducer(s) to control the transcription rate of target genes.

**Figure 1:**
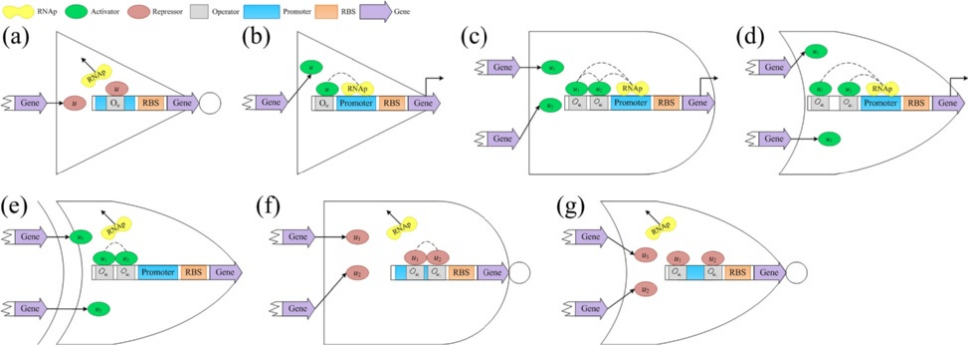
**Expressions of a class of genetic logic gates. (a)** NOT gate; **(b)** Buffer; **(c)** AND gate; **(d)** OR gate; **(e)** XOR gate; **(f)** NAND gate; and **(g)** NOR gate.

For a gene with an operator site which can bind a repressor or activator TF, the promoter activity functions are described as

(2) $$\;f_{\mathrm{NOT}}\left(u\right)=\frac{1}{1+{\left(\frac{u}{K}\right)}^{n}}$$

and

(3) $$\;f_{\mathrm{Buffer}}\left(u\right)=\frac{{\left(\frac{u}{K}\right)}^{n}}{1+{\left(\frac{u}{K}\right)}^{n}}$$

where *f*_NOT_ and *f*_Buffer_ are promoter activity functions for logic NOT and Buffer [26,30], respectively, *n* is the Hill coefficient which denotes the binding cooperativity between TF and the corresponding operator, and *K* is the Hill constant which are proportional to the lengths or affinities of the TF binding sites inserted into the promoter region of the target genes. For logic NOT gate, the input is a repressor and the gene produces a protein only in absence of the repressor; otherwise, the presence of the repressor obstructs the bound of RNAp and promoter. For genetic Buffer, the input is an activator which advances the bound of RNAp and promoter to produce protein. The frameworks for the two logic gates are illustrated in Figures 1(a) and (b), respectively.

For genes with two operator sites, which can bind two repressor TFs or activator TFs, the promoter activity functions are described in accordance with their logic functions as

(4) $$f_{\mathrm{AND}}\left(u_{1},u_{2}\right)=\frac{{\left(\frac{u_{1}}{K_{1}}\right)}^{n_{1}}{\left(\frac{u_{2}}{K_{2}}\right)}^{n_{2}}}{1+{\left(\frac{u_{1}}{K_{1}}\right)}^{n_{1}}+{\left(\frac{u_{2}}{K_{2}}\right)}^{n_{2}}+{\left(\frac{u_{1}}{K_{1}}\right)}^{n_{1}}{\left(\frac{u_{2}}{K_{2}}\right)}^{n_{2}}}$$

(5) $$f_{\mathrm{OR}}\left(u_{1},u_{2}\right)=\frac{{\left(\frac{u_{1}}{K_{1}}\right)}^{n_{1}}+{\left(\frac{u_{2}}{K_{2}}\right)}^{n_{2}}+{\left(\frac{u_{1}}{K_{1}}\right)}^{n_{1}}{\left(\frac{u_{2}}{K_{2}}\right)}^{n_{2}}}{1+{\left(\frac{u_{1}}{K_{1}}\right)}^{n_{1}}+{\left(\frac{u_{2}}{K_{2}}\right)}^{n_{2}}+{\left(\frac{u_{1}}{K_{1}}\right)}^{n_{1}}{\left(\frac{u_{2}}{K_{2}}\right)}^{n_{2}}}$$

(6) $$f_{\mathrm{XOR}}\left(u_{1},u_{2}\right)=\frac{{\left(\frac{u_{1}}{K_{1}}\right)}^{n_{1}}+{\left(\frac{u_{2}}{K_{2}}\right)}^{n_{2}}}{1+{\left(\frac{u_{1}}{K_{1}}\right)}^{n_{1}}+{\left(\frac{u_{2}}{K_{2}}\right)}^{n_{2}}+{\left(\frac{u_{1}}{K_{1}}\right)}^{n_{1}}{\left(\frac{u_{2}}{K_{2}}\right)}^{n_{2}}}$$

(7) $$f_{\mathrm{NAND}}\left(u_{1},u_{2}\right)=\frac{1+{\left(\frac{u_{1}}{K_{1}}\right)}^{n_{1}}+{\left(\frac{u_{2}}{K_{2}}\right)}^{n_{2}}}{1+{\left(\frac{u_{1}}{K_{1}}\right)}^{n_{1}}+{\left(\frac{u_{2}}{K_{2}}\right)}^{n_{2}}+{\left(\frac{u_{1}}{K_{1}}\right)}^{n_{1}}{\left(\frac{u_{2}}{K_{2}}\right)}^{n_{2}}}$$

and

(8) $$f_{\mathrm{NOR}}\left(u_{1},u_{2}\right)=\frac{1}{1+{\left(\frac{u_{1}}{K_{1}}\right)}^{n_{1}}+{\left(\frac{u_{2}}{K_{2}}\right)}^{n_{2}}+{\left(\frac{u_{1}}{K_{1}}\right)}^{n_{1}}{\left(\frac{u_{2}}{K_{2}}\right)}^{n_{2}}}$$

where *f*_AND_, *f*_OR_, *f*_XOR_, *f*_NAND_ and *f*_NOR_ are, respectively, promoter activity functions of logic AND, OR, XOR, NAND and NOR gates, *u*_1_ and *u*_2_ are concentrations of repressor or activator TFs, *K*_1_ and *K*_2_ are Hill constants for *u*_1_ and *u*_2_, respectively, and *n*_1_ and *n*_2_ are the corresponding Hill coefficients. For logic AND, OR and XOR gates, the transcriptional behaviors are regulated by two activator TFs with different binding sites. Two repressor TFs control the genetic expressions of logic NAND and NOR gates. Their construction frameworks are shown in Figure 1(c)-(g).

In [38,39], the promoter and RBS are considered as a promoter-RBS part to regulate the genetic expression because the half-life of mRNA is shorter than the corresponding protein has. One can rewrite (1) as

(9) $${\dot{p}}_{i}=\rho_{i}f_{i}\left(u\right)-\gamma_{i}p_{i}+\rho_{0,i},i=1,\ldots ,L$$

where

$$\rho_{i}=\frac{\alpha_{i}\beta_{i}}{\lambda_{i}},\;\rho_{0,i}=\frac{\alpha_{0,i}\beta_{i}}{\lambda_{i}}$$

Here, *ρ*_*i*_ and *ρ*_0,*i*_ are new synthesis and basal production rates of the protein. The dynamic model of 2*L* differential equation (1) is reduced to the dynamic system with *L* differential equation (9). For real-world implementation, fetching the corresponding promoter-RBS parts from the promoter-RBS library, the synthetic genetic circuit can be realized in the genetic systems.

### Synthetic genetic sequential logic circuits

In digital logic circuits, the output of sequential logic circuits depends not only on the present inputs but also on the past inputs. For synchronous sequential circuits, a clock signal is utilized as a metronome to coordinate actions of circuits, which oscillates between high-level and low-level states. The circuits with triggered clock signals become active either in the rising edge, the falling edge, or in both of the rising and falling edges. For the sequential logic circuit triggered at the rising edge of the clock signal, it becomes active when its clock pulse goes from low to high (0 to 1), and ignores high-to-low (1 to 0) transition.

In genetic logic circuits, oscillation signal produced from a repressilator is not ideal as a clock for use in the kind of circuits relying on the change of rising or falling edge of the clock signal for state transition. Our proposed approach is to introduce the idea of a waveform-shaping circuit in electronics to genetic logic circuits, and reshape the synthesized genetic oscillation signal into a crisp clock signal or a PWM signal with different duty cycles. By regulating the size of duty cycle, the clock pulse can be generated with a rising edge or a falling edge whose frequency is coherent to the oscillation frequency. To use the clock pulse, the designed genetic counter based on the topology of an electronic sequential logic circuit is triggered to generate a clock signal with its frequency is inversely integer multiple to the genetic oscillation.

#### Synthetic genetic oscillator

Oscillation phenomenon in biological systems has been discovered at various levels of biological organization. Its practical function is to control the dosage of drugs or as a synchronous mechanism for cell-cell communication. The oscillation capability depends not only on the network topology but also on the system parameters. Currently, the simplest synthetic genetic oscillator can be synthesized from a single gene repressing itself with a delayed negative feedback loop. An extension of the simplest oscillator, called a repressilator, consists of three genes (*lacI*, *tetR*, *cI*) which represses each other in the cycle chain. The product of the first repressor gene, *lacI* from *E. coli*, inhibits the transcription of the second repressor gene, *tetR* from the tetracycline-resistance transposon Tn10, whose protein product in turn inhibits the expression of the third repressor gene, *cI* from the *λ* phage. Finally, *cI* inhibits *lacI* expression, completing a negative feedback cycle [10]. The dynamic model of the repressilator can be described by

(10) $${\dot{p}}_{i}=\rho_{i}f_{\mathrm{NOT},i}\left(p_{j}\right)-\gamma_{i}p_{i}$$

where *p*_*i*_ and *p*_*j*_ are concentrations of proteins for (*i*, *j*) ≡ (*lacI*, *cI*), (*tetR*, *lacI*) or (*cI*, *tetR*). For other design, the oscillation behavior can be generated by a number of repressor and activator genes in which the number of repressor genes must be odd.

To design the genetic oscillator with desired oscillations, one can realize a gene regulatory network to track a reference sinusoidal signal given by

(11) $$y_{d}=A\sin\left(\omega_{0}t+\varphi \right)+y_{d,0}$$

where *y*_*d*_ is the oscillation signal with the desired amplitude *A*, basal frequency *ω*_0_, phase *φ* and *y*_*d*,0_ is the base level to ensure nonnegative protein concentration. For more details regarding synthetic genetic oscillator design by optimization algorithms one is referred to [35].

#### Waveform-shaping circuit

**Figure 2:**
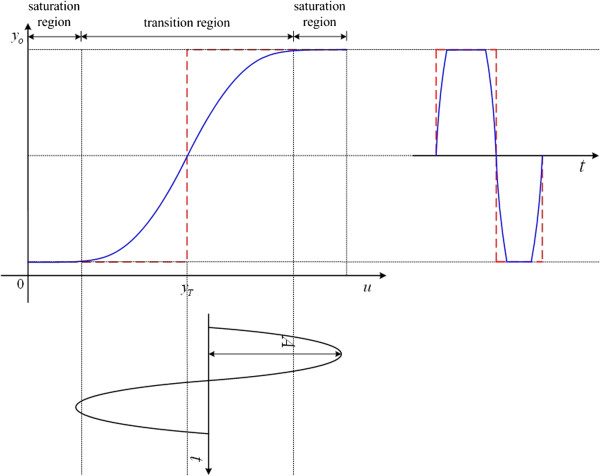
Idea of a genetic waveform shaping circuit.

In electronics, a waveform-shaping circuit is designed to shape the input signal to the desired form according to an input and output (I/O) characteristic curve. For the oscillation input and the clock output, the I/O characteristic curve of the desired waveform-shaping circuit is displayed in Figure 2. A step function (dashed line) with a threshold level *y*_*T*_ is used in electronics. For the input signal with its value larger than the threshold level, it is treated as “logic high”. Otherwise, it is referred to “logic low”. However, in biological systems, an ideal step function doesn’t exist. A sigmoid function (solid line in Figure 2) might be used instead. From the I/O characteristic curve of a sigmoid function, there are two operational regions: saturation and transition. The input signal in the saturation region can be cut-off and hold on the high level or the low level for approximation. In the transition region, the gain in the operation point *y*_*T*_ must be more than (normalized) 1 because it ensures that the input which is larger or less than the threshold level will be amplified or shrunk. By cascading the next sigmoid function, the oscillation input signal will gradually reach the saturation region and remain in the high or low level.

According to this idea, a waveform-shaping circuit can be used to regulate the period of the logic high/low levels of an oscillation signal in a sinusoidal cycle and generate a PWM signal with different duty cycle defined by

(12) $$D=\frac{T_{\mathrm{on}}}{T_{0}}\cdot 100\%$$

where *D* is the duty cycle, *T*_0_ is the basal period of oscillation signal (11) with 2*π*/*ω*_0_ and *T*_on_ being the period of “logic high” in a basal period. For the PWM signals with different duty cycles, the threshold is obtained by considering

(13) $$y_{T}=A\sin\left(\omega_{0}t+\varphi \right)+y_{d,0},t=t_{h}\pm \frac{T_{\mathrm{on}}}{2}$$

with

(14) $$t_{h}=\frac{1}{\omega_{0}}\sin^{-1}\left(1\right)-\frac{\varphi }{\omega_{0}},t_{h}\in \left[\begin{array}{cc} 0 & T_{0} \end{array}\right]$$

**Figure 3:**
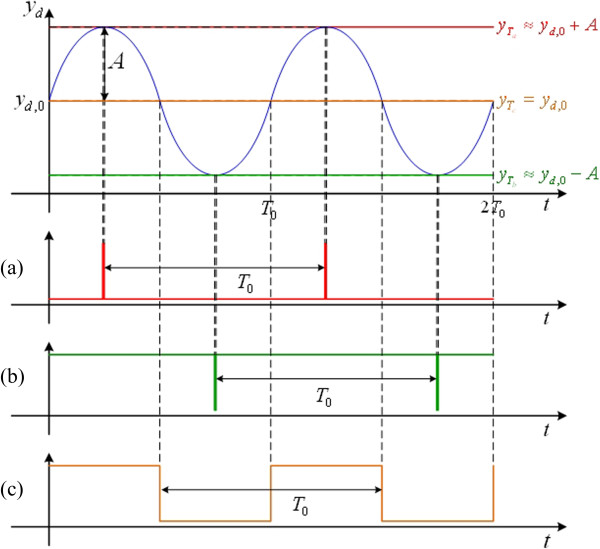
**Ideal PWM signals. (a)** a rising triggered edge; **(b)** a falling triggered edge; and **(c)** 50% duty cycle.

To select the threshold level approaching to *y*_*d*,0_ + *A*, a clock pulse served as a rising triggered edge is generated and shown in Figure 3(a). For the clock pulse regarding as a falling triggered edge shown in Figure 3(b), one can choose the threshold level which is close to *y*_*d*,0_ - *A*. Similarly, a PWM signal with 50% duty cycle, i.e. the clock signal with its frequency is consistent to the genetic oscillator, is synthesized and shown in Figure 3(c) while selecting the base level of the signal *y*_*d*,0_. In other words, the PWM signals with different duty cycles can be synthesized from an oscillation signal via a waveform-shaping circuit in different threshold levels.

#### Realizing a genetic waveform-shaping circuit

In engineered genetic logic circuits, a genetic Buffer [30] is proposed to serve as a buffer between two cascade genetic circuits to enhance logic signal transfer. It’s used here to aid the genetic waveform-shaping circuit design:

(15) $${\dot{p}}_{k}=\rho_{k}f_{\mathrm{Buffer},k}\left(u_{k},K_{k},n_{k}\right)-\gamma_{k}p_{k}+\rho_{0,k},\;k=1,\ldots ,M$$

Its steady-state solution is easily obtained as

(16) $$p_{k,\mathrm{ss}}=\frac{\rho_{k}}{\gamma_{k}}f_{\mathrm{Buffer},k}\left(u_{k},K_{k},n_{k}\right)+\frac{\rho_{0,k}}{\gamma_{k}},\;k=1,\ldots ,M$$

where *p*_*k*_ is the output concentration of the *k*th Buffer, *p*_*k*,*ss*_ denotes its steady-state concentration, *u*_*k*_, *K*_*k*_ and *n*_*k*_ are, respectively, the input concentration, Hill constant, and Hill coefficient of the *k*th Buffer and *ρ*_*k*_, *γ*_*k*_ and *ρ*_0,*k*_ are, respectively, synthesis, decay and basal rates. The second term of the right-hand side of (16) is the minimal level and *ρ*_*k*_/*γ*_*k*_ is the difference between the minimal and maximal levels. Output concentration of the genetic Buffer is the half maximal output concentration when the input concentration equals *K*_*k*_ and thus *K*_*k*_ refers to the threshold level *y*_*T*_.

In each stage, the corresponding inputs and the threshold levels are given by

(17) $$u_{k}=\left\{\begin{array}{l} y_{d},\;k=1\\ p_{k-1},\;1<k\;\leq \;M \end{array}\right)$$

and

(18) $$K_{k}=\left\{\begin{array}{l} y_{T},\;k=1\\ \frac{\rho_{k-1}+\rho_{0,k-1}}{2\gamma_{k-1}},\;1<k\;\leq \;M \end{array}\right)$$

**Figure 4:**
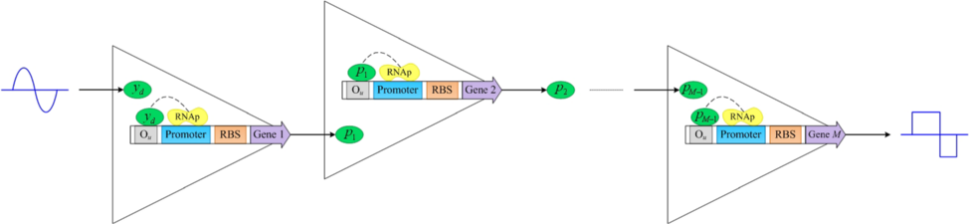
Topology of the designed genetic waveform-shaping circuit.

In the first stage, the input signal is the oscillation signal in (11) and the threshold level is chosen according to the desired duty cycle in (13). For the next stage, the input signal is the output concentration of the previous Buffer and the threshold level is the half maximal output level in the previous one. The topology of our proposed genetic waveform-shaping circuit is displayed in Figure 4. The oscillation signal from protein production of any gene of the repressilator activates the first gene in the genetic waveform-shaping circuit, whose production activates the next gene. Stage by stage, the oscillation can be reshaped to the crisp clock signal or PWM signal. However, the problem of slow convergence to the maximal level is occurred for the larger threshold level *K*_*k*_. To resolve this problem, one can again cascade a Buffer with the design parameters of (16) in the last stage of genetic waveform-shaping circuit to compensate the output level.

The gain at the operating point *K*_*k*_ is obtained by

(19) $$A_{k}={\left(\frac{\partial p_{k,\mathrm{ss}}}{\partial u_{k}}\right|}_{u_{k}=K_{k}}=\frac{\rho_{k}n_{k}}{4\gamma_{k}K_{k}}$$

where *A*_*k*_ is the gain of the *k*th Buffer. The gain is proportional to the Hill coefficient *n*_*k*_ and the synthesis rate *ρ*_*k*_ and is inversely proportional to the Hill constant *K*_*k*_ and the decay rate *γ*_*k*_ at the operating point *u*_*k*_ = *K*_*k*_. To ensure that the necessary condition of the gain at the operating point, *K*_*k*_ should be exceeding 1. At first, one chooses the appropriate Hill constant for the desired synthesized PWM signal and then selects a suitable Hill coefficient *n*_*k*_, synthesis rate *ρ*_*k*_ and decay rate *γ*_*k*_ satisfying (19). From the system parameters in the previous stage, one proceeds to choose the appropriate system parameters in the next stage satisfying (18) and (19). From [38,39], to realize the proposed genetic logic circuit in reality, one can find applicable promoter-RBS components from the constructed promoter-RBS library, whose I/O characteristic curves are capable of satisfying (18) and (19).

#### Design of genetic frequency divider circuit

**Figure 5:**
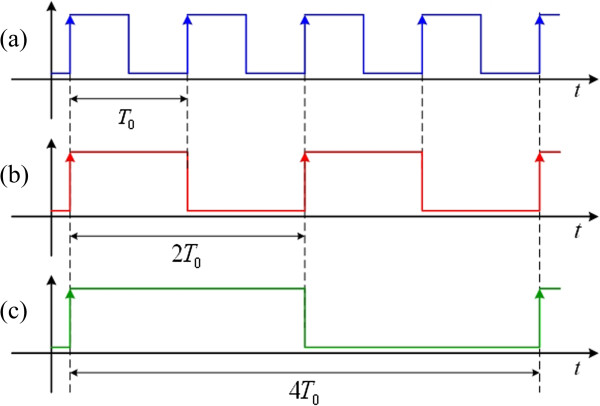
Ideal clock signals with (a) a basal period; (b) double basal period; and (c) quadruple basal period.

Frequency divider in electronics is a device that generates an output signal whose frequency is an inverse multiple to that of the input signal. A sequential logic circuit, counter, is used to achieve this function, which is constructed by a series of flip-flops and triggered by the clock pulse to generate the clock signals with multi-fold basal period. Figure 5 illustrates an ideal clock signal while triggering at the rising edge of the clock signal with the desired basal period.

#### Genetic JK flip-flop

**Figure 6:**
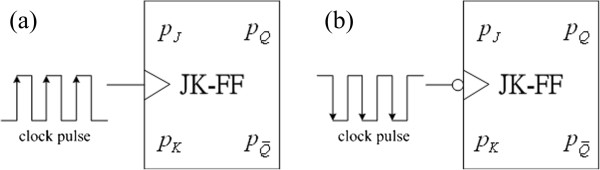
**A class of the clocked genetic JK flip-flops. (a)** a rising edge-triggered one; and **(b)** a falling edge-triggered one.

**Figure 7:**
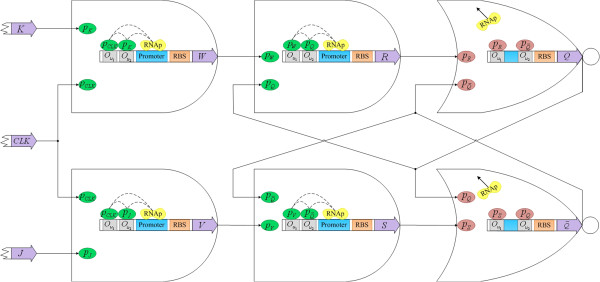
Topology of the rising edge-triggered genetic JK flip-flop.

Genetic JK flip-flops based on the topology of digital logic circuits in electronics divide into the rising edge-triggered one and the falling edge-triggered one shown as in Figure 6. For the rising edge-triggered genetic JK flip-flop, its model is described by

(20) $$\begin{array}{l} {\dot{p}}_{W}=\rho_{W}f_{\mathrm{AND}}\left(p_{K},p_{\mathrm{CLK}},K_{W},n_{W}\right)-\gamma_{W}p_{W},\\ {\dot{p}}_{V}=\rho_{V}f_{\mathrm{AND}}\left(p_{J},p_{\mathrm{CLK}},K_{V},n_{V}\right)-\gamma_{V}p_{V},\\ {\dot{p}}_{R}=\rho_{R}f_{\mathrm{AND}}\left(p_{W},p_{Q},K_{R},n_{R}\right)-\gamma_{R}p_{R},\\ {\dot{p}}_{S}=\rho_{S}f_{\mathrm{AND}}\left(p_{V},p_{\bar{Q}},K_{S},n_{S}\right)-\gamma_{S}p_{S},\\ {\dot{p}}_{Q}=\rho_{Q}f_{\mathrm{NOR}}\left(p_{R},p_{\bar{Q}},K_{Q},n_{Q}\right)-\gamma_{Q}p_{Q},\\ {\dot{p}}_{\bar{Q}}=\rho_{\bar{Q}}f_{\mathrm{NOR}}\left(p_{S},p_{Q},K_{\bar{Q}},n_{\bar{Q}}\right)-\gamma_{\bar{Q}}p_{\bar{Q}} \end{array}$$

where *p*_*CLK*_ is the concentration of clock pulse from low to high, *p*_*W*_, *p*_*V*_, *p*_*R*_, *p*_*S*_, *p*_*Q*_, and $p_{\bar{Q}}$ denote, respectively, the protein concentrations of the genes *W*, *V*, *R*, *S*, *Q* and $\bar{Q}$. The rising edge-triggered genetic JK flip-flop becomes active only when the clock pulse goes from low to high. There are four genetic AND gates and two NOR gates and the topology is displayed in Figure 7. The proteins *p*_*K*_ and *p*_*CLK*_ activate the transcription of the gene *W*. The proteins *p*_*J*_ and *p*_*CLK*_ activate the transcription of the gene *V*. The productions of the genes *W* and *Q* activate the transcription of the gene *R* and the productions of the genes *V* and $\bar{Q}$ activate the transcription of the gene *S*. The proteins *p*_*R*_ and $p_{\bar{Q}}$ inhibit the transcription of the gene *Q* and the proteins *p*_*S*_ and *p*_*Q*_ inhibit the transcription of the gene $\bar{Q}$.

**Figure 8:**
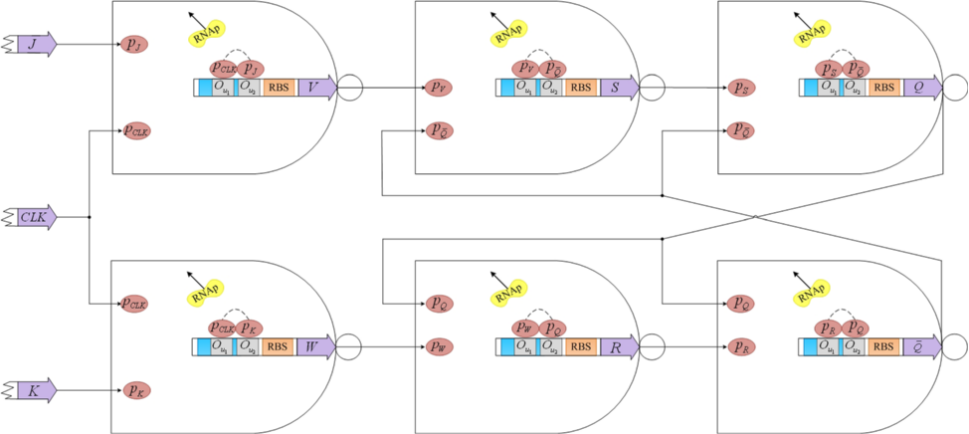
Topology of the falling edge-triggered genetic JK flip-flop.

For the falling edge-triggered genetic JK flip-flop, the model is described by

(21) $$\begin{array}{l} {\dot{p}}_{W}=\rho_{W}f_{\mathrm{NAND}}\left(p_{K},p_{\mathrm{CLK}},K_{W},n_{W}\right)-\gamma_{W}p_{W},\\ {\dot{p}}_{V}=\rho_{V}f_{\mathrm{NAND}}\left(p_{J},p_{\mathrm{CLK}},K_{V},n_{V}\right)-\gamma_{V}p_{V},\\ {\dot{p}}_{R}=\rho_{R}f_{\mathrm{NAND}}\left(p_{W},p_{Q},K_{R},n_{R}\right)-\gamma_{R}p_{R},\\ {\dot{p}}_{S}=\rho_{S}f_{\mathrm{NAND}}\left(p_{V},p_{\bar{Q}},K_{S},n_{S}\right)-\gamma_{S}p_{S},\\ {\dot{p}}_{Q}=\rho_{Q}f_{\mathrm{NAND}}\left(p_{S},p_{\bar{Q}},K_{Q},n_{Q}\right)-\gamma_{Q}p_{Q},\\ {\dot{p}}_{\bar{Q}}=\rho_{\bar{Q}}f_{\mathrm{NAND}}\left(p_{R},p_{Q},K_{\bar{Q}},n_{\bar{Q}}\right)-\gamma_{\bar{Q}}p_{\bar{Q}} \end{array}$$

where *p*_*CLK*_ is the concentration of clock pulse from high to low. This circuit is composed of six genetic NAND gates with its topological structure shown in Figure 8.

#### Genetic counter

**Figure 9:**
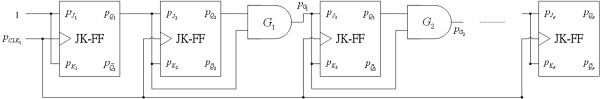
**Topology of the synchronous genetic counter for the clock signals with 2**^***δ***^**-fold basal period.**

To synthesize the clock signal with an inversely multiple of frequency of genetic oscillator, a synchronous genetic counter circuit can be used. The counter circuit in electronics works on the rising or falling edge of the clock and count the number of clock pulses. Based on the feature, one first generates a series of clock pulses using our proposed genetic waveform-shaping circuit, and uses the clock pulse signal to trigger the genetic counter. According to the Karnaugh map in the digital logic theory, the input signals of each genetic JK flip-flop and the topology of genetic counter circuit can be determined. To synthesize the clock signals with 2^*δ*^ -fold basal period in which *δ* is a positive integer, a synchronous genetic counter circuit with *δ* number of rising edge-triggered genetic JK flip-flops is constructed by

(22) $$\begin{array}{l} {\dot{p}}_{W_{1}}=\rho_{W_{1}}f_{\mathrm{AND}}\left(p_{K_{1}},p_{\mathrm{CL}K_{1}},K_{W_{1}},n_{W_{1}}\right)-\gamma_{W_{1}}p_{W_{1}},\\ {\dot{p}}_{V_{1}}=\rho_{V_{1}}f_{\mathrm{AND}}\left(p_{J_{1}},p_{\mathrm{CL}K_{1}},K_{V_{1}},n_{V_{1}}\right)-\gamma_{V_{1}}p_{V_{1}},\\ {\dot{p}}_{R_{1}}=\rho_{R_{1}}f_{\mathrm{AND}}\left(p_{W_{1}},p_{Q_{1}},K_{R_{1}},n_{R_{1}}\right)-\gamma_{R_{1}}p_{R_{1}},\\ {\dot{p}}_{S_{1}}=\rho_{S_{1}}f_{\mathrm{AND}}\left(p_{V_{1}},p_{{\bar{Q}}_{1}},K_{S_{1}},n_{S_{1}}\right)-\gamma_{S_{1}}p_{S_{1}},\\ {\dot{p}}_{Q_{1}}=\rho_{Q_{1}}f_{\mathrm{NOR}}\left(p_{R_{1}},p_{{\bar{Q}}_{1}},K_{Q_{1}},n_{Q_{1}}\right)-\gamma_{Q_{1}}p_{Q_{1}},\\ {\dot{p}}_{{\bar{Q}}_{1}}=\rho_{{\bar{Q}}_{1}}f_{\mathrm{NOR}}\left(p_{S_{1}},p_{Q_{1}},K_{{\bar{Q}}_{1}},n_{{\bar{Q}}_{1}}\right)-\gamma_{{\bar{Q}}_{1}}p_{{\bar{Q}}_{1}},\\ \vdots \\ {\dot{p}}_{W_{\delta }}=\rho_{W_{\delta }}f_{\mathrm{AND}}\left(p_{K_{\delta }},p_{\mathrm{CL}K_{1}},K_{W_{\delta }},n_{W_{\delta }}\right)-\gamma_{W_{\delta }}p_{W_{\delta }},\\ {\dot{p}}_{V_{\delta }}=\rho_{V_{\delta }}f_{\mathrm{AND}}\left(p_{J_{\delta }},p_{\mathrm{CL}K_{1}},K_{V_{\delta }},n_{V_{\delta }}\right)-\gamma_{V_{\delta }}p_{V_{\delta }},\\ {\dot{p}}_{R_{\delta }}=\rho_{R_{\delta }}f_{\mathrm{AND}}\left(p_{W_{\delta }},p_{Q_{\delta }},K_{R_{\delta }},n_{R_{\delta }}\right)-\gamma_{R_{\delta }}p_{R_{\delta }},\\ {\dot{p}}_{S_{\delta }}=\rho_{S_{\delta }}f_{\mathrm{AND}}\left(p_{V_{\delta }},p_{{\bar{Q}}_{\delta }},K_{S_{\delta }},n_{S_{\delta }}\right)-\gamma_{S_{\delta }}p_{S_{\delta }},\\ {\dot{p}}_{Q_{\delta }}=\rho_{Q_{\delta }}f_{\mathrm{NOR}}\left(p_{R_{\delta }},p_{{\bar{Q}}_{\delta }},K_{Q_{\delta }},n_{Q_{\delta }}\right)-\gamma_{Q_{\delta }}p_{Q_{\delta }},\\ {\dot{p}}_{{\bar{Q}}_{\delta }}=\rho_{{\bar{Q}}_{\delta }}f_{\mathrm{NOR}}\left(p_{S_{\delta }},p_{Q_{\delta }},K_{{\bar{Q}}_{\delta }},n_{{\bar{Q}}_{\delta }}\right)-\gamma_{{\bar{Q}}_{\delta }}p_{{\bar{Q}}_{\delta }},\\ {\dot{p}}_{G_{1}}=\rho_{G_{1}}f_{\mathrm{AND}}\left(p_{K_{2}},p_{Q_{2}},K_{G_{1}},n_{G_{1}}\right)-\gamma_{G_{1}}p_{G_{1}},\\ \vdots \\ {\dot{p}}_{G_{\delta -2}}=\rho_{G_{\delta -2}}f_{\mathrm{AND}}\left(p_{K_{\delta -1}},p_{Q_{\delta -1}},K_{G_{\delta -2}},n_{G_{\delta -2}}\right)-\gamma_{G_{\delta -2}}p_{G_{\delta -2}}, \end{array}$$

with the input of each genetic JK flip-flop given by

(23) $$\begin{array}{l} p_{J_{1}}=p_{K_{1}}=1,\\ p_{J_{2}}=p_{K_{2}}=p_{Q_{1}},\\ p_{J_{3}}=p_{K_{3}}=p_{G_{1}},\\ \vdots \\ p_{J_{\delta }}=p_{K_{\delta }}=p_{G_{\delta -2}} \end{array}$$

where $p_{\mathrm{CL}K_{1}}$ is the clock pulse signal from low to high, $p_{Q_{1}}$, $p_{Q_{2}}$, $p_{Q_{\delta }}$ are, respectively, the clock signals with double, quadruple and 2^*δ*^-fold basal periods. Figure 9 shows the topology of the synchronous genetic counter for the clock signals with 2^*δ*^-fold basal period.

**Figure 10:**
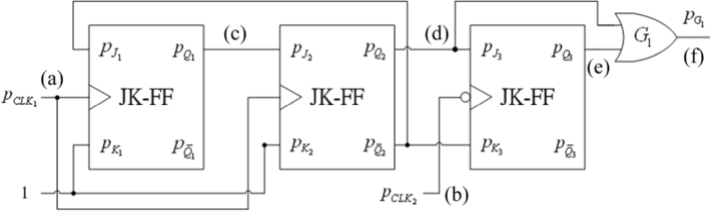
**Topology of the synchronous genetic counter for the clock signal with triple basal period. (a)** Clock pulse signal from low to high; **(b)** Clock pulse signal from high to low; **(c)** Output signal $p_{Q_{1}}$ of the first genetic JK flip-flop; **(d)** Output signal $p_{Q_{2}}$ of the second genetic JK flip-flop; **(e)** Output signal $p_{Q_{3}}$ of the third genetic JK flip-flop; and **(f)** Output signal of logic OR of **(d)** and **(e)**.

**Figure 11:**
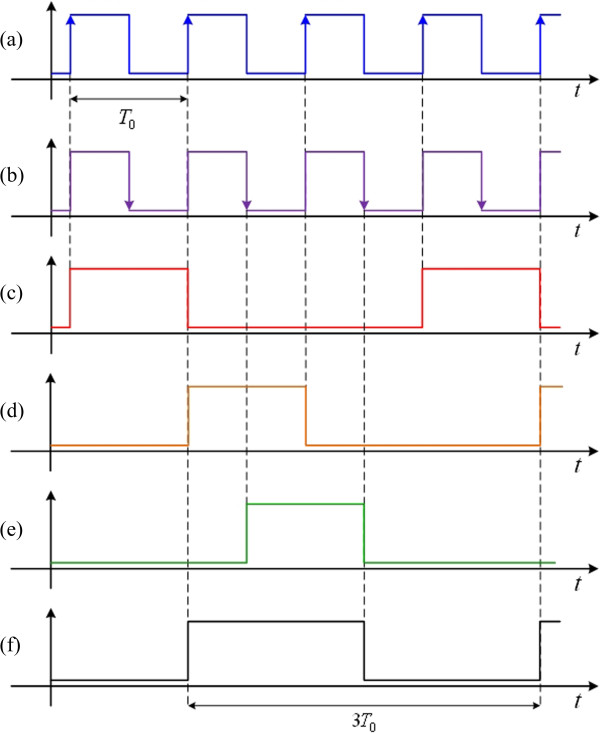
**Ideal signals for synthesizing the clock signal with triple basal period. (a)** Clock pulse signal from low to high; **(b)** Clock pulse signal from high to low; **(c)** Output signal $p_{Q_{1}}$ of the first genetic JK flip-flop; **(d)** Output signal $p_{Q_{2}}$ of the second genetic JK flip-flop; **(e)** Output signal $p_{Q_{3}}$ of the third genetic JK flip-flop; and **(f)** Output signal of logic OR of **(d)** and **(e)**.

To synthesize the clock signal with triple basal period, the synchronous genetic counter with two rising edge-triggered genetic JK flip-flops and a falling edge-triggered genetic JK flip-flop is constructed by

(24) $$\begin{array}{l} {\dot{p}}_{W_{1}}=\rho_{W_{1}}f_{\mathrm{AND}}\left(p_{K_{1}},p_{\mathrm{CL}K_{1}},K_{W_{1}},n_{W_{1}}\right)-\gamma_{W_{1}}p_{W_{1}},\\ {\dot{p}}_{V_{1}}=\rho_{V_{1}}f_{\mathrm{AND}}\left(p_{J_{1}},p_{\mathrm{CL}K_{1}},K_{V_{1}},n_{V_{1}}\right)-\gamma_{V_{1}}p_{V_{1}},\\ {\dot{p}}_{R_{1}}=\rho_{R_{1}}f_{\mathrm{AND}}\left(p_{W_{1}},p_{Q_{1}},K_{R_{1}},n_{R_{1}}\right)-\gamma_{R_{1}}p_{R_{1}},\\ {\dot{p}}_{S_{1}}=\rho_{S_{1}}f_{\mathrm{AND}}\left(p_{V_{1}},p_{{\bar{Q}}_{1}},K_{S_{1}},n_{S_{1}}\right)-\gamma_{S_{1}}p_{S_{1}},\\ {\dot{p}}_{Q_{1}}=\rho_{Q_{1}}f_{\mathrm{NOR}}\left(p_{R_{1}},p_{{\bar{Q}}_{1}},K_{Q_{1}},n_{Q_{1}}\right)-\gamma_{Q_{1}}p_{Q_{1}},\\ {\dot{p}}_{{\bar{Q}}_{1}}=\rho_{{\bar{Q}}_{1}}f_{\mathrm{NOR}}\left(p_{S_{1}},p_{Q_{1}},K_{{\bar{Q}}_{1}},n_{{\bar{Q}}_{1}}\right)-\gamma_{{\bar{Q}}_{1}}p_{{\bar{Q}}_{1}},\\ {\dot{p}}_{W_{2}}=\rho_{W_{2}}f_{\mathrm{AND}}\left(p_{K_{2}},p_{\mathrm{CL}K_{1}},K_{W_{2}},n_{W_{2}}\right)-\gamma_{W_{2}}p_{W_{2}},\\ {\dot{p}}_{V_{2}}=\rho_{V_{2}}f_{\mathrm{AND}}\left(p_{J_{2}},p_{\mathrm{CL}K_{1}},K_{V_{2}},n_{V_{2}}\right)-\gamma_{V_{2}}p_{V_{2}},\\ {\dot{p}}_{R_{2}}=\rho_{R_{2}}f_{\mathrm{AND}}\left(p_{W_{2}},p_{Q_{2}},K_{R_{2}},n_{R_{2}}\right)-\gamma_{R_{2}}p_{R_{2}},\\ {\dot{p}}_{S_{2}}=\rho_{S_{2}}f_{\mathrm{AND}}\left(p_{V_{2}},p_{{\bar{Q}}_{2}},K_{S_{2}},n_{S_{2}}\right)-\gamma_{S_{2}}p_{S_{2}},\\ {\dot{p}}_{Q_{2}}=\rho_{Q_{2}}f_{\mathrm{NOR}}\left(p_{R_{2}},p_{{\bar{Q}}_{2}},K_{Q_{2}},n_{Q_{2}}\right)-\gamma_{Q_{2}}p_{Q_{2}},\\ {\dot{p}}_{{\bar{Q}}_{2}}=\rho_{{\bar{Q}}_{2}}f_{\mathrm{NOR}}\left(p_{S_{2}},p_{Q_{2}},K_{{\bar{Q}}_{2}},n_{{\bar{Q}}_{2}}\right)-\gamma_{{\bar{Q}}_{2}}p_{{\bar{Q}}_{2}}\\ {\dot{p}}_{W_{3}}=\rho_{W_{3}}f_{\mathrm{NAND}}\left(p_{K_{3}},p_{\mathrm{CL}K_{2}},K_{W_{3}},n_{W_{3}}\right)-\gamma_{W_{3}}p_{W_{3}},\\ {\dot{p}}_{V_{3}}=\rho_{V_{3}}f_{\mathrm{NAND}}\left(p_{J_{3}},p_{\mathrm{CL}K_{2}},K_{V_{3}},n_{V_{3}}\right)-\gamma_{V_{3}}p_{V_{3}},\\ {\dot{p}}_{R_{3}}=\rho_{R_{3}}f_{\mathrm{NAND}}\left(p_{W_{3}},p_{Q_{3}},K_{R_{3}},n_{R_{3}}\right)-\gamma_{R_{3}}p_{R_{3}},\\ {\dot{p}}_{S_{3}}=\rho_{S_{3}}f_{\mathrm{NAND}}\left(p_{V_{3}},p_{{\bar{Q}}_{3}},K_{S_{3}},n_{S_{3}}\right)-\gamma_{S_{3}}p_{S_{3}},\\ {\dot{p}}_{Q_{3}}=\rho_{Q_{3}}f_{\mathrm{NAND}}\left(p_{S_{3}},p_{{\bar{Q}}_{3}},K_{Q_{3}},n_{Q_{3}}\right)-\gamma_{Q_{3}}p_{Q_{3}},\\ {\dot{p}}_{{\bar{Q}}_{3}}=\rho_{{\bar{Q}}_{3}}f_{\mathrm{NAND}}\left(p_{R_{3}},p_{Q_{3}},K_{{\bar{Q}}_{3}},n_{{\bar{Q}}_{3}}\right)-\gamma_{{\bar{Q}}_{3}}p_{{\bar{Q}}_{3}}\\ {\dot{p}}_{G_{1}}=\rho_{G_{1}}f_{\mathrm{OR}}\left(p_{Q_{2}},p_{Q_{3}},K_{G_{1}},n_{G_{1}}\right)-\gamma_{G_{1}}p_{G_{1}}, \end{array}$$

with the input of each genetic JK flip-flop given by

(25) $$\begin{array}{l} p_{K_{1}}=p_{K_{2}}=1,\\ p_{J_{1}}=p_{{\bar{Q}}_{2}},p_{J_{2}}=p_{Q_{1}},\\ p_{J_{3}}=p_{Q_{2}},p_{K_{3}}=p_{{\bar{Q}}_{2}} \end{array}$$

where $p_{\mathrm{CL}K_{1}}$ is the clock pulse signal from low to high, $p_{\mathrm{CL}K_{2}}$ is the clock pulse signal from high to low and $p_{G_{1}}$ is the clock signal with triple basal period. The topology of the synchronous genetic counter for the clock signal with triple basal period is displayed in Figure 10 and the corresponding ideal signals are shown in Figure 11.

The above approach is generic, by an analogous way, one is able to determine the corresponding inputs of each genetic JK flip-flop based on the engineering digital logic theory [40] and cascade these basic flip-flips to resemble other types of genetic counters with the desired operational frequency.

## Results

To demonstrate the proposed synthetic genetic sequential logic circuit is effective to realize the function of frequency divider, the following numerical examples are illustrated to confirm the performance of the proposed method.

### Synthetic genetic oscillator

**Figure 12:**
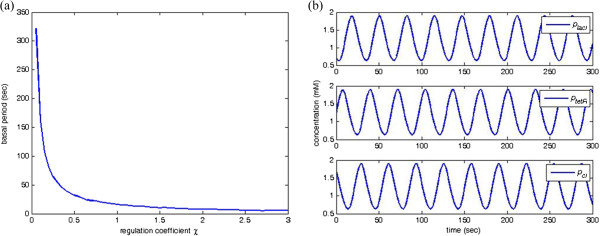
**An example of synthetic genetic oscillator. (a)** Relationship between regulation coefficient *χ* and basal period; and **(b)** Concentration response of genetic oscillator with *χ* = 0.5.

Consider the dynamic model of the synthesized genetic oscillator constructed by three repressive genes given by [34]

(26) $$\begin{array}{l} {\dot{p}}_{\mathrm{lacI}}=\chi \left(0.685\frac{1}{1+p_{\mathrm{cI}}^{4}}-0.233p_{\mathrm{lacI}}\right),p_{\mathrm{lacI}}\left(0\right)=0.7,\\ {\dot{p}}_{\mathrm{tetR}}=\chi \left(0.685\frac{1}{1+p_{\mathrm{lacI}}^{4}}-0.233p_{\mathrm{tetR}}\right),p_{\mathrm{tetR}}\left(0\right)=1.2,\\ {\dot{p}}_{\mathrm{cI}}=\chi \left(0.685\frac{1}{1+p_{\mathrm{tetR}}^{4}}-0.233p_{\mathrm{cI}}\right),p_{\mathrm{cI}}\left(0\right)=1.7 \end{array}$$

where *χ* is the regulation coefficient. The period of oscillation increases when the regulation coefficient *χ* decreases. Otherwise, the period decreases. Figure 12(a) shows the relationship between the basal period and the different regulation coefficient *χ*. Oscillation with the basal period *T*_0_ = 32 sec, the amplitudes *A* = 0.63, and the base level *y*_*d*,0_ = 1.1731 is displayed in Figure 12(b) when *χ* = 0.5.

### Synthesis of PWM signals

**Figure 13:**
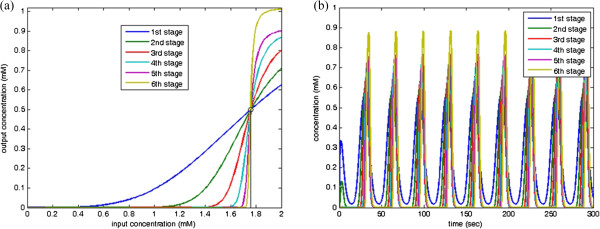
**A PWM signal with *D* = 10%. (a)** I/O characteristic curve in each stage; and **(b)** Concentration responses of the designed clock pulse in each stage.

Suppose protein concentration *p*_*cI*_ is the desired oscillation input. To design the clock pulse signal with *D* = 10%, the designed genetic waveform-shaping circuit is described by

(27) $$\begin{array}{l} \;{\dot{p}}_{1}=f_{\mathrm{Buffer}}\left(p_{\mathrm{cI}},1.76,4\right)-p_{1},\\ \;{\dot{p}}_{2}=f_{\mathrm{Buffer}}\left(p_{1},0.5,4\right)-p_{2},\\ {\dot{p}}_{3}=f_{\mathrm{Buffer}}\left(p_{2},0.5,4\right)-p_{3},\\ {\dot{p}}_{4}=f_{\mathrm{Buffer}}\left(p_{3},0.5,4\right)-p_{4},\\ {\dot{p}}_{5}=f_{\mathrm{Buffer}}\left(p_{4},0.5,4\right)-p_{5},\\ {\dot{p}}_{6}=1.0872f_{\mathrm{Buffer}}\left(p_{5},0.5,4\right)-p_{6} \end{array}$$

where *p*_6_ is the clock pulse signal with *D* = 10%. The circuit has six genetic Buffers. In the first Buffer, the threshold level 1.76 is selected and the corresponding parameters including rate constants, Hill constants, and Hill coefficients and satisfying (18) and (19) are chosen for the second to the fifth Buffers. The last Buffer is to compensate the output of maximal level via choosing an appropriate rate constants in (16). In each stage, the I/O characteristic curves are shown in Figure 13(a) and the output concentrations are shown in Figure 13(b).

**Figure 14:**
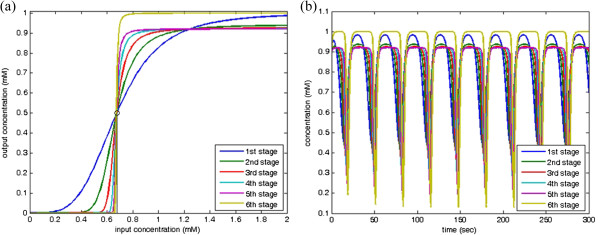
**A PWM signal with *D* = 90%. (a)** I/O characteristic curve in each stage; and **(b)** Concentration responses of the designed clock pulse in each stage.

Similarly, the clock signal with *D* = 90% is generated by the following genetic waveform-shaping circuit

(28) $$\begin{array}{l} {\dot{p}}_{1}=f_{\mathrm{Buffer}}\left(p_{\mathrm{cI}},0.6761,4\right)-p_{1},\\ {\dot{p}}_{2}=f_{\mathrm{Buffer}}\left(p_{1},0.5,4\right)-p_{2},\\ {\dot{p}}_{3}=f_{\mathrm{Buffer}}\left(p_{2},0.5,4\right)-p_{3},\\ {\dot{p}}_{4}=f_{\mathrm{Buffer}}\left(p_{3},0.5,4\right)-p_{4},\\ {\dot{p}}_{5}=f_{\mathrm{Buffer}}\left(p_{4},0.5,4\right)-p_{5},\\ {\dot{p}}_{6}=1.1083f_{\mathrm{Buffer}}\left(p_{5},0.5,4\right)-p_{6} \end{array}$$

where *p*_6_ is the clock pulse signal with *D* = 90%. The first Buffer is designed with the threshold level 0.6761 and the second to the fifth Buffers choose the corresponding threshold level 0.5 satisfying (18). The Buffer in the last stage functions to compensate the output with the maximal level. In each stage, the I/O characteristic curves are shown in Figure 14(a) and the output concentrations are shown in Figure 14(b).

**Figure 15:**
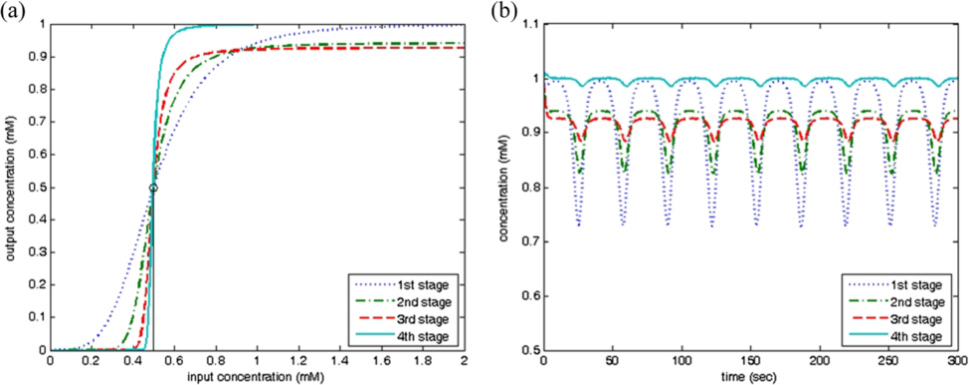
**A PWM signal with *D* = 100%. (a)** I/O characteristic curve in each stage; and **(b)** Concentration responses of the designed DC signal in each stage.

According the proposed approach, the oscillation signal can be rectified and filtered as a direct current (DC) output. The genetic waveform-shaping circuit with *D* = 100% is described by

(29) $$\begin{array}{l} {\dot{p}}_{1}=f_{\mathrm{Buffer}}\left(p_{\mathrm{cI}},0.5,4\right)-p_{1},\\ {\dot{p}}_{2}=f_{\mathrm{Buffer}}\left(p_{1},0.5,4\right)-p_{2},\\ {\dot{p}}_{3}=f_{\mathrm{Buffer}}\left(p_{2},0.5,4\right)-p_{3},\\ {\dot{p}}_{4}=1.0851f_{\mathrm{Buffer}}\left(p_{3},0.5,4\right)-p_{4}, \end{array}$$

where *p*_4_ is the DC signal with *D* = 100%. There are four genetic Buffers. In the first Buffer, the threshold level 0.5 is chosen and the second to fifth Buffers choose the threshold level 0.5. The Buffer in the last stage functions to compensate the output of maximal level. In each stage, the I/O characteristic curves are shown in Figure 15(a) and the output concentrations are shown in Figure 15(b).

### Synthesis of clock signals

**Figure 16:**
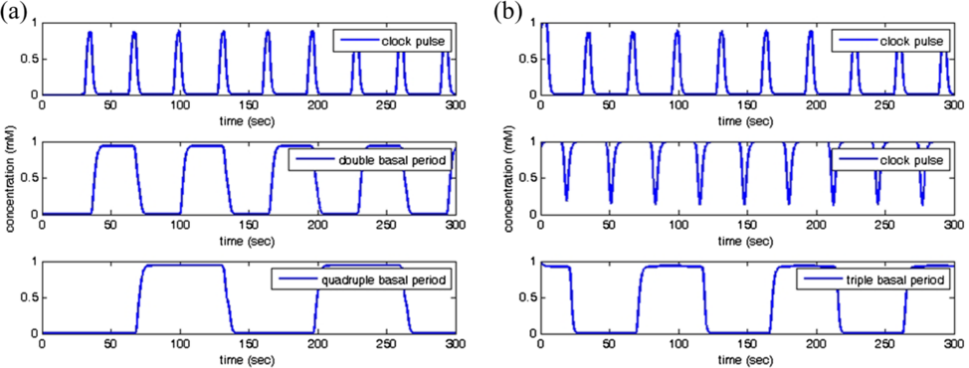
**Response of the designed clock signals. (a)** double and quadruple basal periods; and **(b)** triple basal period.

To design the clock signals with the double and the quadruple basal periods, we suppose *δ* = 2, all synthesis and decay rates are 1, all Hill constants are 0.5, all Hill coefficients are 4 in (22), $p_{\mathrm{CL}K_{1}}$ is the clock pulse generated from (27) and the high level DC signal is synthesized by (29). The concentration responses of the designed clock signals with double and quadruple basal periods are shown in Figure 16(a). State change of the genetic JK flip-flop occurs when the clock pulse goes from 0 to 1. For the clock signal with triple basal period, suppose that all variables remain the same to the above example, $p_{\mathrm{CL}K_{1}}$ and $p_{\mathrm{CL}K_{2}}$ are, respectively, the clock pulses generated in (27) and (28), and the DC signal is synthesized by (29). Concentration response of the designed clock signal with the triple basal period is successfully generated, shown as in Figure 16(b).

## Discussion

The goal of synthetic biology is to design genetic circuits with specific functions by using the approaches of mathematics and engineering. Several genetic logic gates have recently been developed and experimentally realized [16-22]. In [16-18], simplified schemes of various genetic logic gates have been constructed by using basic biological components such as activator/repressor genes, reporter gene, promoter and RBS. For a gene with multiple binding sites, the transcription rate of the gene can be regulated by the same number of regulated proteins. The modified protease can control the degradation rate of protein [19]. For logic NOT gate, the gene with a repressor regulated protein can generate the I/O characteristic curves with an inverse sigmoid function. A complementary form of NOT gate is “Buffer” [30] which is regulated by an activator protein to reshape the I/O characteristic curves for enhancing sharpness of a biologic response with a sigmoidal function. For logic AND gate with two binding sites, the genetic expression can be regulated by two activator proteins. In [22], the activator proteins HrpR and HrpS control the promoter of the corresponding reporter gene to realize logic AND gate. By cascading a NOT gate in the outputs of AND/OR gates, the NAND/NOR gates can be synthesized [20-22]. In [22], a reporter gene with different promoters (lac, BAD and lux) and different RBS parts (rbs30, rbs33, rbsH, etc) can determine the values of Hill constant and coefficient and achieve the different NOT gates with different threshold levels. Similarly, one also can choose different promoter and RBS components with repressor or activator proteins to construct the corresponding system parameters for other logic gates. Through synchronous cascades of these genetic logic gates based on the topology of electronic circuits, the toggle switch and oscillator have been characterized in real world by a class of Hill differential equations, and realized in *Escherichia coli*[9,10]. The previous papers have exhibited the possibility of realization of a class of fundamental biological devices in real world which form a basis for implementing more complicated combinational or sequential biological circuits. To realize the proposed genetic waveform-shaping circuit and genetic counter using a systematic approach, we first can identify the different promoter-RBS parts to achieve the basic logic gates, and then assemble these logic gates with the topologies in Figures 4, 7, 8, 9, and 10.

Reporter protein or green fluorescent protein (GFP) has been used in real-world experiments to reflect genetic expression [41]. By using a flow cytometer, the intensity of fluorescence of GFP is measurable. The system parameters in the dynamic model of genetic circuits can be identified from these measurement data using system identification methods [22]. In [38,39], a class of robust genetic circuits has been constructed by selecting the applicable promoter-RBS component from a promoter-RBS library. Therefore, to realize our proposed genetic logic circuits including the genetic waveform-shaping circuit and genetic counter, one first establishes the measurement device which exhibits fluorescence concentrations of a series of a repressor or activator gene with different promoter-RBS components and TFs via fluorescence measurement [41] and then rebuild a promoter-RBS library with information of system parameters in terms of our mathematical model describing the behaviors of genetic logic gates by using the system identification methods [30,35]. To select the adequate promoter-RBS component generating the I/O characteristic curves of Buffers [30] and satisfying the designed conditions (18) and (19) from the rebuilt promoter-RBS library via an optimization algorithm (such as [35]), the clock pulse signals can be synthesized based on the proposed cascaded genetic logic circuit. Similarly, one can also choose the suitable promoter-RBS components to realize the various genetic logic gates and assemble these logic gates based the proposed topology. The designed genetic counters can be triggered by the synthesized clock pulse signals to generate the clock signals with multi-basal periods.

In sequential logic circuits, the triggered signals could be rising or falling edge of a clock signal. One can use clock pulse signals to replace the rising or falling edge to trigger the sequential logic circuits such as our proposed genetic counters. The proposed genetic counters with a function of frequency divider based on the topology of electronic circuits can be used to generate clock signals with multi-basal period. In other words, one can utilize the proposed approach to generate clock signals whose periods is an integer multiple of 24-hour from a cell with circadian rhythm.

## Conclusions

This paper has proposed a synthetic genetic sequential logic circuit as a frequency divider. The synthesized clock frequency is inversely multiple to that of the genetic oscillator which generates the fundamental sine wave. Through controlling threshold level of the genetic Buffer, the proposed waveform-shaping circuit regulates time duration of logic high/low levels in a basic sinusoidal cycle for an oscillation input and generates ideal pulse signals with the coherent frequency to the genetic oscillator. Regarding the generated pulse signal as rising/falling edge of the clock signal with base frequency, a genetic synchronous counter circuit based on the topology of digital sequential logic circuit is triggered by a pulse wave form to synthesize a clock signal with the inverse multiple frequency to the genetic oscillator. Experimental results *in silico* show the synthesizing genetic clock with double, triple, and quadruple basal periods while operating based on a genetic oscillator. By extending the proposed design principle, a class of multi-basal period clock signals can be generated in a straightforward manner.

## Competing interests

The authors declare that they have no competing interests.

## Authors’ contributions

CLL formulated the research topic. CHC developed the method, performed the simulation and wrote the manuscript. Both authors read and approved the manuscript.
